# Review on bisphenol A and the risk of polycystic ovarian syndrome: an insight from endocrine and gene expression

**DOI:** 10.1007/s11356-022-19244-5

**Published:** 2022-02-24

**Authors:** Risani Mukhopadhyay, Navya B. Prabhu, Shama Prasada Kabekkodu, Padmalatha S. Rai

**Affiliations:** 1 Department of Biotechnology, Manipal School of Life Sciences, Manipal Academy of Higher Education, Manipal, India; 2 Department of Cell and Molecular Biology, Manipal School of Life Sciences, Manipal Academy of Higher Education, Manipal, India

**Keywords:** Polycystic ovarian syndrome, Bisphenol A, Hormones, Gene expression, Steroidogenesis, Gonadotropins

## Abstract

Bisphenol A (BPA) is one of the most widely studied endocrine disrupting chemicals because of its structural similarity to 17-β estradiol; its ability to bind as an agonist/antagonist to estrogen receptors elicits adverse effects on the functioning of the metabolic and endocrinal system. Therefore, BPA has been thoroughly scrutinized concerning its disruption of pathways like lipid metabolism, steroidogenesis, insulin signaling, and inflammation. This has resulted in reports of its correlation with various aspects of cardiovascular diseases, obesity, diabetes, male and female reproductive disorders, and dysfunctions. Among these, the occurrence of the polycystic ovarian syndrome (PCOS) in premenopausal women is of great concern. PCOS is a highly prevalent disorder affecting women in their reproductive age and is clinically characterized by hyperandrogenism, ovulatory dysfunction, and polycystic ovarian morphology, along with metabolism-related dysfunctions like hyperinsulinemia, obesity, and insulin resistance. In this review, we analyzed certain researched effects of BPA, while focusing on its ability to alter the expression of various significant genes like *GnRH*, *AdipoQ*, *ESR1*, *StAR*, *CYP11A1*, *CYP19A1*, and many more involved in the pathways and endocrine regulation, whose disruption is commonly associated with the clinical manifestations of PCOS.

## Introduction

As time passes and human needs evolve, there is a demand for the modernization of industries. This causes high production rates of various chemicals. Some of these chemicals may have harmful effects on the body of both humans and animals. One such class of chemicals is known as endocrine-disrupting chemicals (EDCs). They are a heterogeneous group of molecules that are of both synthetic and natural origin and possess the ability to mimic or antagonize natural hormones and can interact with hormone receptors (Yilmaz et al. 2020).

Among various types of EDCs, the most abundantly available chemical is bisphenol A (BPA). It is a white, crystalline solid compound of synthetic nature, which serves as a precursor to polycarbonates, polyesters, polysulfones, polyether ketones, and a major class of epoxy resins (vinyl ester resins). Due to its properties, it has been classified as a plasticizer. Hence, it has an extensive range of applications and is commonly found in baby bottles, metal-based food, beverage can linings, ophthalmic lenses, medical and dental supplies, electronics and electric appliances, water pipes, and carbonless receipt (Yilmaz et al. 2020). Since BPA is small in structure and is lipophilic in nature, it gives it the ability to cross cellular barriers and bio-accumulate in adipose tissues of most animals and humans, thereby accommodating its participation in various reactions that take place in the bod**y** (Rutkowska and Rachoń 2014).

BPA has been identified as a xenoestrogen, because of its ability to disturb the functioning of the endocrine system by mimicking the behavior of the natural estrogen, 17-β estradiol (Iso et al. 2006). Its activity was defined towards classical nuclear ER_α_ and ER_β_ receptors, their expression, interactions, and effect on steroidogenesis. Due to such disruptions, the subsequent effects observed are in the growth of ovarian follicles, enlarged prostate, increased bodyweight, alterations in testosterone excretion, and sperm quality (Melzer et al. 2011; Richter et al. 2007).

BPA is observed to show moderately acute toxicity in animals, especially mammals. It has been reported to show effects on the functioning of other hormones like androgens, insulin, prolactin, and thyroid hormone (Wetherill et al. 2007; Renaud et al. 2019). The most common pathological effects observed in laboratory studies with and peer-reviewed human studies are obesity, cardiovascular diseases, hyperinsulinemia, thyroid, hypertension, ovarian and testicular developmental issues, polycystic ovarian syndrome (PCOS), and cancer **(**Michałowicz 2014). BPA tends to affect various pathways like the insulin pathway, glucose pathway, lipid metabolism, protein metabolism, and ovarian steroidogenesis, thereby having the potential to cause metabolic-endocrine disorders like PCOS in premenopausal women. PCOS is an extremely prevalent metabolic endocrine disorder in premenopausal women, displaying a broad spectrum of clinical manifestations and subsequent associative morbidities (Belenkaia et al. 2019). It is a heterogeneous disorder that is characterized by a combination of symptoms and manifestations of excess androgen in the form of hyperandrogenism and/or hirsutism, ovarian dysfunction both morphological and endocrinal, and menstrual irregularities observed as the number of cycles per year or the length of each cycle.

The worldwide prevalence of PCOS is estimated to range from 4 to 12% (Meier 2018) making it one of the most common endocrine-metabolic disorders in women during their reproductive age. Despite this, PCOS continues to be one of the poorly understood medical disorders among, patients, practitioners, pharmaceuticals, and even the research community (Escobar-Morreale 2018). Over the decades, research on PCOS has suggested that it is multifaceted in nature and there are various factors like genetic (gene interactions and effect of environment on gene expression), epigenetic (modifications and alterations), endocrine, metabolic, environmental factors, and lifestyle that influence the onset of PCOS in women (Prabhu et al. 2021).

## Human interaction with BPA

Exposure in humans can be classified into two broad categories, environmental exposure and intake from food. The possible routes of exposure are ingestion, inhalation, and dermal uptake in humans. Environmental exposure is due to various factors such as BPA leaching into the atmosphere or water bodies by industrial activities, dumping of waste in landfills, and many more. Whereas the exposure due to intake of food is related to the interaction of plants and animals with BPA, the consequent bioaccumulation, and the contact of food with packaging materials (Kang et al. 2006). Unlike most EDCs, BPA has a short half-life of 6 h and has the ability to get eliminated as BPA-glucuronide due to enzyme activity under normal conditions. Yet, it is considered to be persistent because of its widespread application and continuous exposure of the population to it (Encarnação et al. 2019). Therefore, to set a safety level of exposure to humans, the US Environmental Protection Agency (EPA) and the National Toxicology Program (NTP) convened a committee to study the various biological effects that occur due to typical human exposure. After various experimental studies, the levels reported are as follows, the no-observed-adverse-effects level (NOAEL) is 5.0 mg/kg body-weight/day and the low-observed-adverse-effects level (LOAEL) is observed to be at 50 mg/kg body-weight/day. Therefore, consequently, the tolerable daily intake (TDI) of BPA was reported to be 4 µg/kg body-weight/day (Hong and Yang 2017).

## Metabolism and toxicity of BPA

**Fig. 1:**
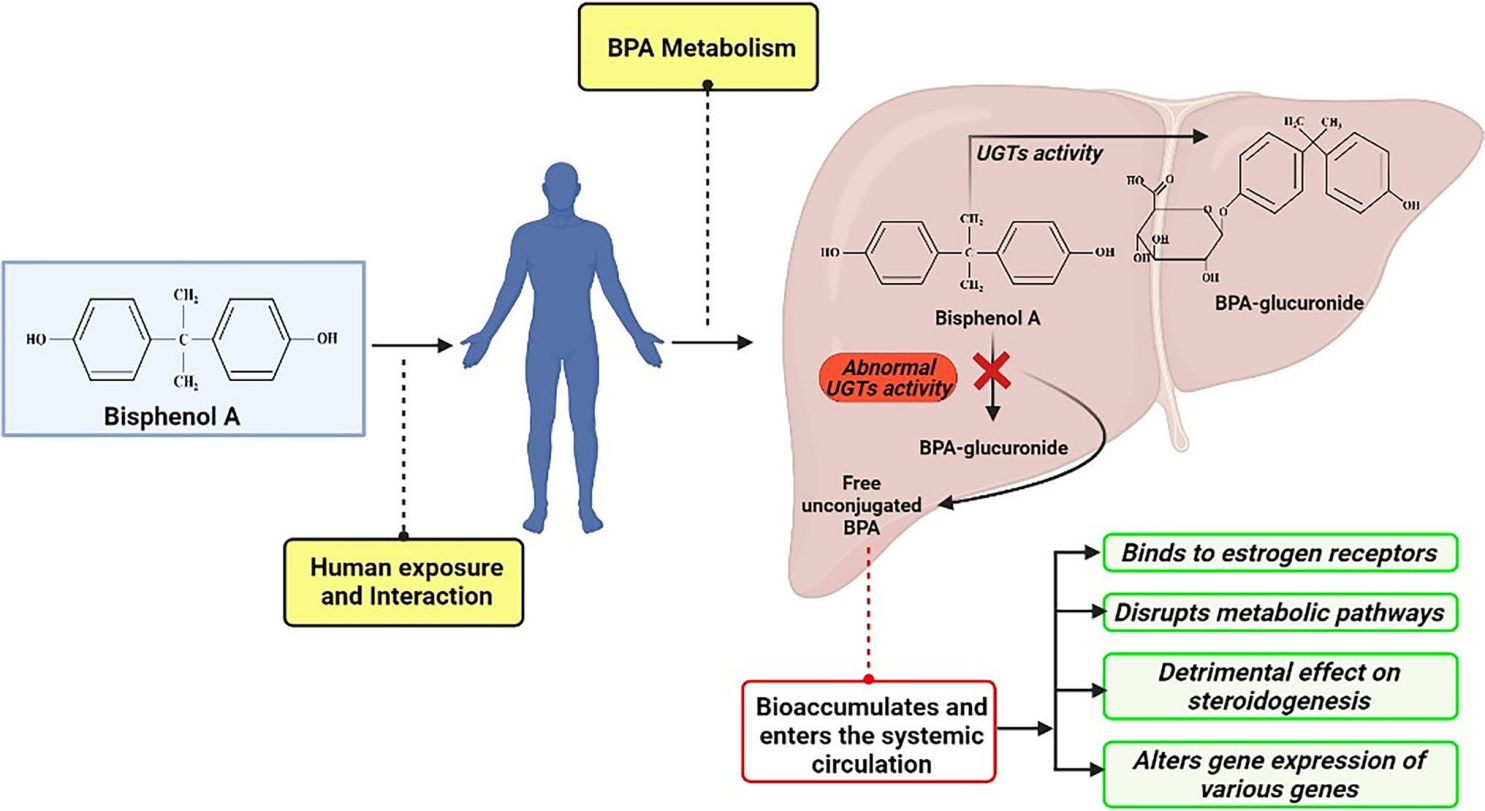
Human exposure to BPA and its consequent metabolism

The primary organ responsible for the metabolism of BPA in humans is the liver. In the liver, phase II conjugation of BPA results in the transformation to mainly BPA-glucuronide (BPA-G) and small amounts of BPA-sulfate (BPA-S) conjugates (Fan et al. 2017). Uridine-5-diphospho-glucuronosyltransferases (UGTs) are the important class of enzymes involved in the catalysis of BPA glucuronidation that results in the transformation of BPA to BPA-G, and the main enzymes involved in the process are believed to be hepatic UGT2B15 and UGT1A9 (Hanioka et al. 2008). Furthermore, BPA is reported to be majorly excreted in the urine as glucuronide (94.6%) (Provencher et al. 2014). The abnormalities in the functioning of UGTs enzyme cause the increase in levels of unconjugated BPA concentration in the system (Khan et al. 2021). Alongside, its ability to mimic the behavior of 17-*β* estradiol (Iso et al. 2006) results in the disruption of various pathways, causing moderately acute toxicity in humans. The most common pathological effects observed in laboratory studies with, and peer-reviewed human studies are obesity, cardiovascular diseases, hyperinsulinemia, thyroid, hypertension, ovarian and testicular developmental issues, PCOS, and cancer (Michałowicz 2014) (Fig. 1).

This review attempts to correlate the alteration in expression of various genes targeted by BPA and endocrine function in the pathophysiology of PCOS. It focuses on gene expression and endocrinal regulation in animal models and in vitro cultures of human cell lines, because of the limitations like uncontrolled exposure, the impact of various other lifestyle factors, and the ethical constraint observed in human studies.

## Search strategies

The search was conducted using PubMed as an electronic database. Studies were identified using the combination of the following search terms: “bisphenol A” AND “gene expression” OR “mRNA expression” OR “altered expression” OR “polycystic ovarian syndrome” OR “female reproductive system” OR “Insulin resistance” OR “Hyperandrogenism” OR “Ovulatory dysfunction” OR “estrous cyclicity” OR “Chronic inflammation” OR “steroidogenic hormones” OR “gonadotropins” OR “female sex hormones.” All the data from both animal and in vitro studies on the relationship among BPA and the different aspects of the female reproductive and endocrine system, along with the clinical manifestation of PCOS were considered for inclusion. Furthermore, the information regarding mRNA expression, alteration of steroid hormone levels, BPA exposure, and PCOS were discussed in the review.

## Gene expression studies

BPA has been observed to target and alter the gene expression of several important genes associated with PCOS, and their corresponding protein level dysregulation supports the observation (Table 1). Many observational studies have researched the alteration in gene expression by evaluating the levels of their transcribed mRNA, in various aspects like the disruption of the hypothalamic-pituitary-ovary (HPO) axis, disruption of steroidogenic and metabolic pathways and their results can be associated with the pathophysiology and manifestations of PCOS (Table 2).

**Table 1:** List of genes targeted by BPA in the female reproductive system

Sl. no	Gene	Gene symbol	Role	Reference
1	*Kisspeptin*	*KISS1*	Codes for the hypothalamic kisspeptin protein involved in the regulation of pubertal development and estrogen-mediated negative feedback of gonadotropin -releasing hormone	Dungan et al. (2006)
2	*Gonadotropin releasing hormone 1*	*GNRH1* (humans) Gnrh1 (mice)	Codes for the preproprotein that generates peptides that stimulate the secretion of gonadotropins, i.e., luteinizing hormone (by GNRH1) and follicle-stimulating hormone	Wang et al. (2014)
3	*Gonadotropin releasing hormone 2*	*GNRH2* (humans) *gnrh2* (fish)		Qin et al. (2012)
4	*Resistin*	*RETN*	Codes for the preprotein of the Resistin hormone, which is an adipose-derived hormone that participates in an inflammatory response	Menale et al. (2017)
5	*Adiponectin*	*AdipoQ*	Codes for a protein hormone involved in the regulation of glucose levels and the breakdown of fatty acids	Menale et al. (2017)
6	*Leptin*	*LEP*	Codes for an adipose-derived hormone that regulates appetite and fat storage in adipocytes	Ariemma et al. (2016)
7	*Interleukin 6*	*IL-6*	Codes for an interleukin that can be a pro-inflammatory cytokine and have an anti-inflammatory action in muscles	Ariemma et al. (2016)
8	*Interferon gamma*	*IFNG*	Codes for a pro-inflammatory cytokine that plays an important role in both innate and adaptive immunity, by stimulating the action of macrophages, natural killer cells, and neutrophils	Ariemma et al. (2016)
9	*Tumor necrosis factor-alpha*	*TNFA*	Codes for a pro-inflammatory cytokine produced during inflammation by macrophages or monocytes, signal for cellular events that lead to necrosis or apoptosis	Ariemma et al. (2016)
10	*Insulin-like growth factor 1*	*IGF1*	Codes for a protein that has a similar structure to Insulin and is responsible for growth stimulation in all cell types in adults	Aluru et al. (2010)
11	*Insulin-like growth factor 2*	*IGF2*	Codes. for a protein that has a similar structure to Insulin and is responsible for growth stimulation in all cell types in fetal development	Aluru et al. (2010)
12	*Insulin-like growth factor 1 receptor*	*IGF1R (Ra and Rb)*	Codes for a transmembrane receptor that belongs to the class of tyrosine kinase receptors and plays an important role in growth	Aluru et al. (2010)
13	*Estrogen receptor 1*	*ESR1*	Codes for ER α i.e., an estrogen receptor activated by estradiol that triggers cell proliferation in reproductive tissues and can translocate into the nuclease to regulate the activity of various genes by binding to the DNA	Wang et al. (2014)
14	*Estrogen receptor 2*	*ESR2*	Codes for ER β i.e., an estrogen receptor activated by estrogen that may inhibit cell proliferation in reproductive tissues and has the tumor-suppressing ability, by translocating into the nuclease and regulating the activity of various genes by binding to the DNA	Wang et al. (2014)
15	*Luteinizing Hormone/Choriogonadotropin Receptor*	*LHCGR*	Codes for a receptor protein called the luteinizing hormone/chorionic gonadotropin receptor, that as a receptor for two ligands: luteinizing hormone and a similar hormone called chorionic gonadotropin and allows the body to respond appropriately to these hormones	Xi et al. (2011)
16	*Steroidogenic Acute Regulatory Protein*	*StAR*	Codes for a transport protein that regulates cholesterol transfer within the mitochondria	Zhou et al. (2008)
17	*Follicle Stimulating Hormone Receptor*	*FSHR*	Codes for a transmembrane receptor that interacts with FSH	Xi et al. (2011)
18	*Follicle Stimulating Hormone subunit beta*	*FSHB*	Codes for the β-subunit of FSH protein that coupled with the common alpha subunit form the FSH protein. With LH, it induces egg and sperm production	Xi et al. (2011)
19	*Luteinizing Hormone subunit beta*	*LHB*	Codes for the β-subunit of LH protein that coupled with the common alpha subunit from the LH protein. With FSH, it induces egg and sperm production	Xi et al. (2011)
20	*Cytochrome P450 Family 11 Subfamily A Member 1*	*CYP11A1*	Codes for the enzyme CYP450-side-chain cleavage (p450scc), that catalyzes the conversion of Cholesterol to Pregnenolone	Zhou et al. (2008)
21	*Cytochrome P450 family 19 subfamily A member 1*	*CYP19A1*	Codes for Aromatase, the enzyme that catalyzes the formation of estrogens from androgens	Zhou et al. (2008)
22	*Cytochrome P450 family 17 subfamily A member 1*	*CYP17A1*	Codes for p450c17 enzyme that both hydroxylates pregnenolone at the 17^th^ carbon and lysates 21-carbon steroids to 19-carbon steroids	Zhou et al. (2008)
23	*Hydroxy-delta-5-steroid dehydrogenase, 3 beta and steroid delta-isomerase 1*	*HSD3B1*	Codes for the isomerase, 3 β-Hydroxysteroid dehydrogenase that is responsible for the conversion of pregnenolone to progesterone and DHEA to androstenedione, etc	Zhang et al. (2018)
24	*Anti-Mullerian hormone*	*AMH*	Codes for the preprotein of Anti-Mullerian hormone that inhibits the response of granulosa cell to FSH and LH	Li et al. (2014)
25	*Estrogen Related Receptor Gamma*	*ESRRG*	Codes for a protein that binds to the estrogen response element and steroidogenic factor 1 response element, to activate genes controlled by them and has been known to function as a transcriptional activator of DNA cytosine-5-methyltransferases 1 (DNMT1) expression	Arase et al. (2011)
26	*Hydroxysteroid 17-beta dehydrogenase*	*HSD17B (B1, B2, B3)*	Codes for the isomerase, 17 β-hydroxysteroid dehydrogenase that regulate the levels of sex steroids by reducing the C-17 hydroxy group of androgens and estrogens	Shi et al. (2021)
27	*Peroxisome proliferator-activated receptor gamma*	*PPARG*	Codes for the protein PPAR-γ that activate genes stimulating the uptake of lipids and adipogenesis in fat cells, thereby regulating the storage of fatty acid and glucose metabolism	Ariemma et al. (2016)
28	*Insulin*	*INS*	Codes for the hormone Insulin, which controls the glucose levels in the blood by regulating the metabolism of carbohydrates and promoting the absorption of glucose by the liver, adipocytes, and skeletal muscle cells	Haq et al. (2020)

*ER *α estrogen receptor α, *ER* *β* estrogen receptor β, *FSH* follicle−stimulating hormone, *LH* luteinizing hormone, *DHEA* dehydroepiandrosterone, *PPAR−* γ peroxisome proliferator−activated receptor gamma

**Table 2:** Alteration in gene expression on BPA exposure

Sl. no	Gene	Study model	Dosage of BPA	Alteration in gene expression	Reference
1	*KISS1*	Female Sprague Dawley rats	0.05, 0.5 mg kg^-1^ day^-1^	Downregulated	Qiu et al. (2020)
			10 mg kg^-1^ day^-1^	Upregulated	
		ICR mice	20 μg kg^-1^ day^-1^	Upregulated (only in AVPV)	Wang et al. (2014)
		CD-1 mice	12, 25, 50 mg kg^-1^ day^-1^	Upregulated	Xi et al. (2011)
2	*GNRH1*	ICR mice	20 μg kg^-1^ day^-1^	Upregulated	Wang et al. (2014)
		Embryonic mouse hypothalamus cell line N44 (mHypoE-N44)	200 μM	Downregulated	Warita et al. (2013)
		CD-1 mice	12, 25, 50 mg kg^-1^ day^-1^	Upregulated	Xi et al. (2011)
		Wistar rats	0.5 mg kg^-1^	Upregulated	Monje et al. (2010)
			20 mg kg^-1^	Downregulated	
		Female Sprague Dawley rats	0.05, 0.5 mg kg^-1^ day^-1^	Downregulated	(Qiu et al. (2020)
			10 mg kg^-1^ day^-1^	Upregulated	
3	*GNRH2*	*Gobiocypris rarus*	5 μg L^-1^	Downregulated	Qin et al. (2012)
			15 μg L^-1^	Upregulated	
4	*RETN*	Human (In vitro culture of differentiated adipocytes)	1, 10, 100 nM	Upregulated	Menale et al. (2017)
5	*AdipoQ*	Human (In vitro culture of differentiated adipocytes)	1nM	Upregulated	Menale et al. (2017)
			10, 100 nM	Downregulated	
		Human (In vitro culture of adipose tissue)	10 nM	Downregulated	Ahmed et al. (2020)
		Mouse (In vitro culture of 3T3-L1 pre-adipocytes)	1 nM	Upregulated	Ariemma et al. (2016)
		Human (In vitro culture of mature adipocytes and SVF cells)	0.1 nM	Downregulated	Cimmino et al. (2019)
6	*LEP*	Mouse (In vitro culture of 3T3-L1 pre-adipocytes)	1 nM	Upregulated	Ariemma et al. (2016)
		Human (In vitro culture of mature adipocytes and SVF cells)	0.1 nM	Upregulated	Cimmino et al. (2019)
7	*IL6*	Mouse (In vitro culture of 3T3-L1 pre-adipocytes)	1 nM	Upregulated	Ariemma et al. (2016)
		Human (In vitro culture of adipose tissue)	10, 10^4^ nM	Downregulated	Ahmed et al. (2020)
		Human (In vitro culture of mature adipocytes and SVF cells)	0.1 nM	Upregulated	Cimmino et al. (2019)
8	*IFNG*	Mouse (In vitro culture of 3T3-L1 pre-adipocytes)	1 nM	Upregulated	Ariemma et al. (2016)
		F344 rats	4, 40, 400 mg kg^-1^ day^-1^	Downregulated	Miao et al. (2008)
9	*TNFA*	F344 rats	4, 40, 400 mg kg^-1^ day^-1^	Downregulated	Miao et al. (2008)
		Human (In vitro culture of adipose tissue)	1 nM	Downregulated	Ahmed et al. (2020)
		Mouse (In vitro culture of 3T3-L1 pre-adipocytes)	1 nM	Upregulated	Ariemma et al. (2016)
10	*IGF1*	*Oncorhynchus mykiss* (oocytes)	30, 100 μg mL^-1^	Downregulated	Aluru et al. (2010)
11	*IGF2*	*Oncorhynchus mykiss* (oocytes)	30, 100 μg mL^-1^	Downregulated	Aluru et al. (2010)
12	*IGF1R (Ra and Rb)*	*Oncorhynchus mykiss* (oocytes)	30, 100 μg mL^-1^	Downregulated	Aluru et al. (2010)
		Human (BG-1 ovarian adenocarcinoma cell line)	10^-5^ M	Upregulated	Kang et al. (2013)
13	*ESR1*	ICR mice	20 μg kg^-1^ day^-1^	Upregulated	Wang et al. (2014)
		Human (BG-1 ovarian adenocarcinoma cell line)	10^-5^ M	Upregulated	Kang et al. (2013)
		F344 rats	4, 40, 400 mg kg^-1^ day^-1^	Upregulated	Miao et al. (2008)
		*Gobiocypris rarus*	15 μg L^-1^	Downregulated	Zhang et al. (2018)
14	*ESR2*	ICR mice	20 μg kg^-1^ day^-1^	Upregulated	Wang et al. (2014)
		CD-1 mice	12, 25, 50 mg kg^-1^ day^-1^	Upregulated	Xi et al. (2011)
		*Gobiocypris rarus*	15 μg L^-1^	ESR2 α—No effects ESR2 β – Upregulated	Zhang et al. (2018)
15	*LHCGR*	Zebrafish ovarian follicle cells	5 μM	Upregulated	Liu et al. (2013)
		CD-1 mice	12 mg kg^-1^ day^-1^	Downregulated	Xi et al. (2011)
			25, 50 mg kg^-1^ day^-1^	Upregulated	
		*Catla catla* (ovary tissue)	10, 100, 1000 μg L^-1^	Upregulated	Faheem et al. (2017)
16	*StAR*	CD-1 mice	12, 50 mg kg^-1^ day^-1^	Upregulated	Xi et al. (2011)
			25 mg kg^-1^ day^-1^	Downregulated	
		Human (In vitro culture of ovarian granulosa KGN cells)	0.5, 5, 50, 500 μg L^-1^	Downregulated	Shi et al. (2021)
		*Catla catla* (ovary tissue)	10, 100, 1000 μg L^-1^	Upregulated	Faheem et al. (2017)
		*Gobiocypris rarus*	15 μg L^-1^	Downregulated	Zhang et al. (2018)
		CD-1 mice (antral follicles)	10, 100 μg mL^-1^	Downregulated	Peretz and Flaws (2013)
		Wistar rats (In vitro culture of granulosa cells)	0.1, 1, 10 μM	Downregulated	Samardzija et al. (2018)
			50, 100 μM	Upregulated	
			10^-4^ M	Upregulated	Zhou et al. (2008)
17	*FSHR*	CD-1 mice	12 mg kg^-1^ day^-1^	Downregulated	Xi et al. (2011)
			25, 50 mg kg^-1^ day^-1^	Upregulated	
		*Catla catla* (ovary tissue)	10, 100, 1000 μg L^-1^	Upregulated	Faheem et al. (2017)
18	*FSHB*	CD-1 mice	12, 25, 50 mg kg^-1^ day^-1^	Upregulated	Xi et al. (2011)
19	*LHB*	CD-1 mice	12, 25 mg kg^-1^ day^-1^	Upregulated	Xi et al. (2011)
			50 mg kg^-1^ day^-1^	Downregulated	
20	*CYP11A1*	CD-1 mice	12, 25, 50 mg kg^-1^ day^-1^	Upregulated	Xi et al. (2011)
		CD-1 mice (antral follicles)	10, 100 μg mL^-1^	Downregulated	Peretz and Flaws (2013)
		Sprague Dawley rat (ovary)	10^-4^ M	Downregulated	Zhou et al. (2008)
			10^-5^ M	Upregulated	
		Mouse urogenital sinus	20 μg kg^-1^	Upregulated	Arase et al. (2011)
		Wistar rats (In vitro culture of granulosa cells)	100 μM	Upregulated	Samardzija et al. (2018)
		Human (placental JEG-3 cell lines)	1-1000 nM	Downregulated	Chu et al. (2018)
21	*CYP19A1*	CD-1 mice	12, 25, 50 mg kg^-1^ day^-1^	Upregulated	Xi et al. (2011)
		Human (In vitro culture of ovarian granulosa KGN cells)	0.5, 5, 50, 500 μg L^-1^	Upregulated	Shi et al. (2021)
		*Catla catla* (ovary tissue)	10, 100, 1000 μg L^-1^	Upregulated	Faheem et al. (2017)
		Sprague Dawley rat (ovary)	10^-4^10^-6^ M	Downregulated	Zhou et al. (2008)
		Mouse urogenital sinus	20 μg kg^-1^	Upregulated	Arase et al. (2011)
		Human (placental JEG-3 cell lines)	11000 nM	Downregulated	Chu et al. (2018)
		Human (ovarian granulosa-like (KGN) cell lines)	50 μM	Downregulated	Watanabe et al. (2012)
		*Gobiocypris rarus*	5, 15 μg L^-1^	Downregulated	Qin et al. (2012)
22	*CYP17A1*	CD-1 mice	12, 25 mg kg^-1^ day^-1^	Downregulated	Xi et al. (2011)
			50 mg kg^-1^ day^-1^	Upregulated	
		Sprague Dawley rat (ovary)	10^-4^ M	Upregulated	Zhou et al. (2008)
		*Gobiocypris rarus*	13.75±1.63 μg L^-1^	Downregulated	Zhang et al. (2017)
23	*HSD3B1*	*Gobiocypris rarus*	15 μg L^-1^	Upregulated	Zhang et al. (2018)
		Wistar rats (In vitro culture of granulosa cells)	100 μM	Upregulated	Samardzija et al. (2018)
24	*AMH*	Mice	10, 40, 60 mg kg^-1^	Upregulated	Li et al. (2014)
		Mice	5, 50, 500 μg kg^-1^	Downregulated	Cao et al. (2018)
25	*ESRRG*	Mouse urogenital sinus	20 μg kg^-1^	Upregulated	Arase et al. (2011)
26	*HSD17B (B1, B2, B3)*	Human (In vitro culture of ovarian granulosa KGN cells)	0.5, 5, 50, 500 μg L^-1^	Downregulated	Shi et al. (2021)
27	*PPARG*	Mouse (In vitro culture of 3T3-L1 pre-adipocytes)	1 nM	Upregulated	Ariemma et al. (2016)
28	*INS*	Wistar rats	50, 500, 2500, 5000 μg kg^-1^	Upregulated	Haq et al. (2020)

*KISS1* Kisspeptin, *GNRH1* gonadotropin−releasing hormone 1, *GNRH2* gonadotropin−releasing hormone 2, *RETN* resistin, *AdipoQ* adiponectin, *LEP* leptin, *IL−6* interleukin 6, *IFNG* interferon gamma, *TNFA* tumor necrosis factor−alpha, *IGF1* insulin−like growth factor 1, *IGF2* insulin−like growth factor 2, *IGF1R* insulin−like growth factor 1 receptor, *ESR1* estrogen receptor 1, *ESR2* estrogen receptor 2, *LHCGR* luteinizing hormone/choriogonadotropin receptor, *StAR* steroidogenic acute regulatory protein, *FSHR* follicle−stimulating hormone receptor, *FSHB* follicle−stimulating hormone subunit beta, *LHB* luteinizing hormone subunit beta, *CYP11A1* cytochrome P450 family 11 subfamily A member 1, *CYP19A1* cytochrome P450 family 19 subfamily A member 1, *CYP17A1* cytochrome P450 family 17 subfamily A member 1, *HSD3B1* hydroxy−delta−5−steroid dehydrogenase, 3 beta and steroid delta−isomerase 1, *AMH* anti−Mullerian hormone, *ESRRG* estrogen−related receptor gamma, *HSD17B* hydroxysteroid 17−beta dehydrogenase, *PPARG* peroxisome proliferator−activated receptor gamma, *INS* insulin, μg microgram, kg kilogram, mg milligram, μM micromolar, nM nanomolar, L litre, mL mililiter, M molar

## Gonadotropin dysregulation

In female animals, the estrous cycle and in female humans the menstrual cycle is regulated by the interaction between hormones released by the hypothalamus, pituitary, and ovaries, thereby forming the HPO-axis. The pulsatile release of gonadotrophin-releasing hormone (GnRH) from the hypothalamus, prompts the release of luteinizing hormone (LH) and follicle-stimulating hormone (FSH) from the anterior segment of the pituitary. These gonadotrophs in the ovarian level, act on ovarian follicles to release progesterone (P) and estrogen (E). Estradiol (E2) is one of three estrogens produced, that regulates the release of LH and GnRH by a negative feedback action (Adachi et al. 2007) on binding with estrogen receptor α (ER α) exhibited by kisspeptin neurons in the arcuate nucleus (ARC) and anteroventral periventricular nucleus (AVPV) (Kinoshita et al. 2005). E2 decreases the expression of *KISS1* mRNA in ARC and increases *KISS1* mRNA in AVPA, resulting in negative and positive feedback respectively (Dungan et al. 2006). As BPA possesses the ability to mimic the action of E2, it can bind to ER α (Fang et al. 2000), and thereby disrupt the functioning of the hypothalamus and pituitary gland. Also, the expression of genes like *KISS1*, *GNRH*, *LHB*, and *FSHB* has been subjected to alteration, as observed in various animal and in vitro studies when exposed to BPA.

The effects of BPA highly depend on the time of exposure, its duration, and dosage, hence having varying impacts on the result as observed in various studies. The dose-dependent exposure of mouse embryonic hypothalamic cells to BPA for 3 h, displayed significantly reduced expression of *Gnrh1* at 200μM when compared with control and other lower doses. The results suggested that *Gnrh1* expression in the cells was unresponsive to treatment below 200μM and its downregulation at high dose exposure results in disruption of maturation of the HPO-axis (Warita et al. 2013). The downregulation of the gene allows the hypothesis that high dose exposure to BPA incapacitates female rats from producing normal LH surges as seen in Wistar rats (Monje et al. 2010), since the gene codes for GnRH which stimulates the secretion of gonadotropins like LH and FSH from the pituitary gland.

On the other hand, neonatal exposure to BPA in female Sprague Dawley rats is seen to cause an upregulation of *KISS1* and *GNRH1* genes. Kisspeptin regulates the surge in gonadotropin levels during the initiation of female puberty and its activation by the neonatal BPA exposure shows that it can affect pubertal development (Qiu et al. 2020). This observation is also seen in a study conducted by Xi et al. (2011) on CD-1 mice, perinatal exposure to BPA causes upregulation of *KISS1* mRNA and *GNRH* mRNA in adults, which in turn causes alteration in the gene expression of gonadotropins (*FSHB*, *LHB*) and their receptors (*FSHR*, *LHCGR*). As the feedback system is disturbed, these changes in the hypothalamus and pituitary can be both the cause and the consequence of the altered HPO-axis functioning.

The upregulation of *ESR1* (Wang et al. 2014) and *ESR2* (Xi et al. 2011; Wang et al. 2014) mRNA in the pituitary exhibit a molecular basis of the selective action of BPA on the AVPV-kisspeptin neuron, which causes further upregulation of *GNRH* as observed in a study on ICR mice when exposed to 20 μg/kg body weight per day of BPA (Wang et al. 2014). The dose-dependent alteration in expression of GnRH is not only observed in mammals but also in teleost (Qin et al. 2012).

## Disrupted ovarian steroidogenesis and hyperandrogenism

Ovarian steroidogenesis is a culmination of the interaction between LH, FSH, and enzymes like p450scc, p450c17, and aromatase. Androgen formation in ovarian theca cells is stimulated by LH and in granulosa cells, and biosynthesis of E2 from androgens is stimulated by FSH. StAR protein regulates the transfer of cholesterol into granulosa cells so that p450scc can convert it to pregnanolone, which is then converted to progesterone by HSD-3 *β* and simultaneously to androstenedione by the 17,20-lyase activity of p450c17; furthermore, progesterone also gets converted to androstenedione in the theca cells. This androstenedione gets converted to estrone by aromatase and further to E2 by the activity of HSD-17 *β* in granulosa cells (Hannon and Flaws 2015). The dysregulation of gene expression caused by BPA exposure, at the hypothalamus and pituitary level impacts the functioning of the ovary. The upregulation of *FSHB*, *LHB* (Xi et al. 2011) *FSHR*, and *LHCGR* (Xi et al. 2011; Faheem et al. 2017) genes on high-dose BPA exposure and its consequent increase in stimulation of the ovarian follicles positively correlate it with the altered expression of genes coding for enzymes that participate in the conversion of androgens to E2 (Xi et al. 2011).

A study conducted by Zhou et al. (2008) on Sprague Dawley rats treated with varying doses of BPA exhibited upregulation of *StAR*, *CYP11A1*, and *CYP17A1* in ovarian T-1 cells. At a 10^–4^ M dose of BPA, the increased expression of *StAR* and *CYP11A1* may promote the production of more progesterone which paired with the upregulation of *CYP17A1* at 10^–5^ M results in increased production of androgens. The sudden downregulation of *CYP17A1* at 10^−4^ M is assumed to be the result of cytotoxicity of granulosa cells from continuous testing (Zhou et al. 2008). The downregulation of *CYP19A1* suggests that there is a decrease in the conversion of androgen to E2, on exposure to BPA (Xi et al. 2011; Chu et al. 2018; Watanabe et al. 2012). On exposure to BPA, the upregulation of *HSD3B1* (Wang et al. 2014; Samardzija et al. 2018) and downregulation of *HSD17B* gene (Shi et al. 2021) further consolidate the chances of increased androgen levels, as there is a possible increase in conversion of progesterone to androgens and androstenedione to E2, respectively.

Furthermore, BPA exposure has also been observed to upregulate the genes encoding orphan nuclear receptors like estrogen-related receptor-γ (ESRR-γ) (Arase et al. 2011), thereby suggesting that BPA can participate in an orphan nuclear receptor-mediated alteration of the expression of steroidogenic enzymes in the ovary (Xi et al. 2011). The contradicting and differing results of alteration in gene expression could be credited to the difference in cell types and organisms under study, their dosage, and time of dosage. The disruption caused by BPA in the expression of genes participating in ovarian steroidogenesis could cause hyperandrogenism and ovulatory dysfunction (Rutkowska and Rachoń 2014).

## Ovarian folliculogenesis disruption

The dysregulation of gonadotropin secretion and its consequent disruption of the HPO-axis contribute to ovarian follicular arrest and major changes in the morphology and functioning of the ovary. The upregulation of *LHB* (Xi et al. 2011) may contribute to the hypertrophy of ovarian follicles thereby leading to their premature luteinization (Azziz et al. 2016). It is observed that on pre-pubertal exposure to high doses of BPA, the expression of *AMH* increases in ovarian granulosa cells. The upregulation of *AMH* can be contributed to the effect of BPA in increased numbers of small antral follicles in comparison to the overall numbers of follicles present in the ovary (Li et al. 2014). This could lead to a decrease in FSH sensitivity, which could further impair follicular growth (Gruijters et al. 2003).

Whereas exposure to low doses of BPA shows downregulation of the *AMH* gene, this result is hypothesized to be caused by the effect of BPA on the reduction of granulosa cell activity and its subsequent accelerated apoptosis (Cao et al. 2018). Another possible factor could be the downregulation of *IGF1*, *IGF2*, and *IGF1R* genes on perinatal BPA exposure, which tends to result in the lack of development of ovarian cells and suppresses growth in general (Aluru et al. 2010).

## Adipose tissue dysfunction, insulin resistance, and chronic inflammation

Adipose tissue acts not only as a fat storage reservoir that participates in homeostasis but also as an endocrine organ that secretes regulatory adipokines, cytokines, and chemokines. BPA is known to disrupt various metabolic pathways such as lipid metabolism, carbohydrate metabolism, insulin signaling pathway, and cause adipose tissue inflammation. A study conducted by Ariemma et al. (2016) observed the various effects of prolonged low dose exposure of BPA on adipocyte differentiation. BPA exposure tends to increase pre-adipocyte growth by upregulation of *PPARG*, *AdipoQ*, and *LEP* genes that regulate adipogenesis and paired with the proinflammatory action of BPA; this exacerbates insulin sensitivity in adipocytes and hampers the insulin signaling pathway (Ariemma et al. 2016). It is observed that the regulation of glucose level on BPA exposure compensates for the upregulation of the *INS1* gene (Haq et al. 2020). On exposure to BPA, the upregulation of *LEP* (Ariemma et al. 2016; Cimmino et al. 2019) in cultures of adipocytes displays a possibility of an increase in fat storage, thereby drawing a correlation between BPA exposure and obesity.

Furthermore, the dose-dependent downregulation of *AdipoQ* mRNA in in vitro cultures of adipocytes (Cimmino et al. 2019; Menale et al. 2017; Ahmed et al. 2020) suggests the dysregulation in the breakdown of fatty acids and desensitization of cells to insulin. This combined with the upregulation of *RETN* (Menale et al. 2017) inhibits insulin action and presents a possible link between BPA exposure causing obesity and insulin resistance. As adipose tissue insulin resistance is intricately linked to increased inflammation, the alteration in expression of inflammatory cytokines such as *IL6* (Ariemma et al. 2016; Cimmino et al. 2019; Ahmed et al. 2020), *IFNG* (Ariemma et al. 2016; Miao et al. 2008), *TNFA* (Miao et al. 2008) that are adipose-derived confirms the effect of BPA exposure leading to chronic inflammation in adipocytes may cause insulin resistance.

## Hormonal changes caused by BPA

The alteration in the expression of hormone-coding genes and enzymes brings about biochemical changes in hormone and enzyme levels. Since BPA can have post-translational effects on the expression of proteins, it is important to corroborate the results of gene expression with protein expression (Table 3).

**Table 3:** Hormone level alterations observed in animal models

Sl. no	Organisms	Treatment dosage	Hormones	Hormone level (mean/mean ± standard deviation)		Results	Reference
				Treatment group	Control group		
1	Female Sprague Dawley rats	6.2 – 2.5 mg kg^−1^ bodyweight (BPA50)	LH	1.89 ng mL^-1^	2.44 ng mL^-1^	BPA exposure lowered GnRH-induced LH and disrupted estrus cyclicity in the BPA500 group	Fernández et al. (2009)
		62.5—25.0 mg kg^−1^ bodyweight (BPA500)	LH	1.57 ng mL^-1^	2.44 ng mL^-1^		
2	Female Sprague Dawley rats	0.625 mg kg^−1^ body weight (BPA5)	E2	14 pg mL^-1^	16 pg mL^-1^	1. Neonatal exposure to BPA alters sex hormone levels in adult rats 2. Adult BPA500 and BPA50 animals had higher levels of T and E2, and all BPA groups showed lower levels of P than controls, although BPA500 was the most affected group	Fernández et al. (2010)
			T	270 pg mL^-1^	260 pg mL^-1^		
			P	25 ng mL^-1^	31 ng mL^-1^		
		6.25 mg kg^−1^ body weight (BPA50)	E2	21 pg mL^-1^	16 pg mL^-1^		
			T	495 pg mL^-1^	260 pg mL^-1^		
			P	23.5 ng mL^-1^	31 ng mL^-1^		
		62.5 mg kg^−1^ body weight BPA500)	E2	20.5 pg mL^-1^	16 pg mL^-1^		
			T	450 pg mL^-1^	260 pg mL^-1^		
			P	16.6 ng mL^-1^	31 ng mL^-1^		
3	Female Sprague Dawley rats	10^–7^ M	E2	58 ng mL^-1^	60 ng mL^-1^	1. Significant lower E2 levels were observed in all the BPA-treated groups compared to the control 2. Significant higher T levels were observed in all the BPA-treated groups compared to the control 3. Significantly higher P levels were observed in all the BPA-treated groups compared to control, except in the 10^–4^ M group where it decreased significantly	Zhou et al. (2008)
			T	0.021 ng mL^-1^	0.015 ng mL^-1^		
			P	2.7 ng mL^-1^	2.35 ng mL^-1^		
		10^–6^ M	E2	46 ng mL^-1^	60 ng mL^-1^		
			T	0.02 ng mL^-1^	0.015 ng mL^-1^		
			P	2.7 ng mL^-1^	2.35 ng mL^-1^		
		10^–5^ M	E2	34 ng mL^-1^	60 ng mL^-1^		
			T	0.2225 ng mL^-1^	0.015 ng mL^-1^		
			P	3.1 ng mL^-1^	2.35 ng mL^-1^		
		10^–4^ M	E2	26 ng mL^-1^	60 ng mL^-1^		
			T	0.036 ng mL^-1^	0.015 ng mL^-1^		
			P	1.9 ng mL^-1^	2.35 ng mL^-1^		
4	SPF C57BL/6 female mice	5 μg kg^-1^ bodyweight	E2	33.47 ± 3.96 ng mL^-1^	38.02 ± 2.84 pg mL^-1^	Serum E2 and AMH levels were decreased in the exposed groups in comparison to the control group	Cao et al. (2018)
			AMH	15.29±2.04 ng mL^-1^	17.72 ± 2.53 ng mL^-1^		
		50 μg kg^-1^ bodyweight	E2	37.50 ± 6.07 pg mL^-1^	38.02 ± 2.84 pg mL^-1^		
			AMH	16.30 ± 2.28 ng mL^-1^	17.72 ± 2.53 ng mL^-1^		
		500 μg kg^-1^ bodyweight	E2	34.42±3.75 pg mL^-1^	38.02 ± 2.84 pg mL^-1^		
			AMH	16.09 ± 1.92 ng mL^-1^	17.72 ± 2.53 ng mL^-1^		
5	Female Wistar rats	3 μg kg^-1^ day^-1^	LH	5.75 ng mL^-1^	3 ng mL^-1^	1. Serum levels of LH and E2 show a significant increase in the BPA-treated group when compared to the control group 2. Though serum levels of FSH remain unchanged	Gámez et al. (2015)
			FSH	120 ng mL^-1^	120 ng mL^-1^		
			E2	11.5 pg mL^-1^	8.5 pg mL^-1^		
6	Female mice	10 mg kg^-1^	E2	74.38 pmol L^-1^	74.65 pmol L^-1^	1. The serum levels of E2 decreased with an increase in the dose of BPA exposure. But the difference is not significant 2. The serum P4 levels significantly decreased with an increase in the dose of BPA	Li et al. (2014)
			P	18.85 nmol L^-1^	43.65 nmol L^-1^		
		40 mg kg^-1^	E2	59.4 pmol L^-1^	74.65 pmol L^-1^		
			P	10.54 nmol L^-1^	43.65 nmol L^-1^		
		160 mg kg^-1^	E2	72.65 pmol L^-1^	74.65 pmol L^-1^		
			P	12.93 nmol L^-1^	43.65 nmol L^-1^		
7	Female Sprague Dawley rat	50 μg kg^-1^ (BPA1)	T	0.39 ± 0.04 ng mL^-1^	0.34 ± 0.01 ng mL^-1^	1. Plasma concentrations of T increase with the increase of dose in all BPA treated groups, but the difference is not significant 2. E2, P, and LH concentrations show a significant decline in BPA3 and BPA4 when compared with the control 3. Plasma concentrations of FSH decreases in BPA4, but the difference in low treatment groups is not significant	Ijaz et al. (2020)
			E2	1.99 ± 010 pg mL^-1^	1.30 ± 0.06 pg mL^-1^		
			P	0.62 ± 0.02 ng mL^-1^	0.56 ± 0.03 ng mL^-1^		
			LH	2.33 ± 0.12 ng mL^-1^	2.64 ± 0.04 ng mL^-1^		
			FSH	4.44 ± 0.05 ng mL^-1^	4.68 ± 0.10 ng mL^-1^		
		500 μg kg^-1^ (BPA2)	T	0.40 ± 0.04 pg mL^-1^	0.34 ± 0.01 ng mL^-1^		
			E2	1.84 ± 0.04 pg mL^-1^	1.90 ± 0.06 pg mL^-1^		
			P	0.50 ± 0.02 ng mL^-1^	0.56 ± 0.03 ng mL^-1^		
			LH	2.31 ± 0.02 ng mL^-1^	2.64 ± 0.04 ng mL^-1^		
			FSH	4.43 ± 0.04 ng mL^-1^	4.68 ± 0.10 ng mL^-1^		
		5 mg kg^-1^ (BPA3)	T	0.65 ± 0.04 ng mL^-1^	0.34 ± 0.01 ng mL^-1^		
			E2	1.77 ± 0.5 pg mL^-1^	190 ± 0.06 pg mL^-1^		
			P	0.35 ± 0.02 ng mL^-1^	0.56 ± 0.03 ng mL^-1^		
			LH	2.24 ± 0.2 ng mL^-1^	2.64 ± 0.04 ng mL^-1^		
			FSH	4.42 ± 0.05 ng mL^-1^	4.68 ± 0.10 ng mL^-1^		
		50 mg kg^-1^ (BPA4)	T	1.58 ± 0.07 ng mL^-1^	0.34 ± 0.01 ng mL^-1^		
			E2	1.12 ± 0.06 pg mL^-1^	1.90 ± 0.06 pg mL^-1^		
			P	0.36 ± 0.02 ng mL^-1^	0.56 ± 0.03 ng mL^-1^		
			LH	2.20 ± 0.01 ng mL^-1^	2.64 ± 0.04 ng mL^-1^		
			FSH	4.18 ± 0.04 ng mL^-1^	4.68 ± 0.10 ng mL^-1^		

*BPA* bisphenol A, *GnRH* gonadotropin−releasing hormone, *LH* luteinizing hormone, *E2* estradiol, *T* testosterone, *P* progesterone, *AMH* anti−Mullerian hormone, *FSH* follicle−stimulating hormone, mL milliliter, pmol picomole, nmol nanomole, pg picogram, ng nanogram, L liter

The study conducted by Zhou et al. (2008) on female Sprague Dawley rats showed elevated levels of testosterone and progesterone, along with reduced levels of estradiol. The levels are a result of the altered gene expression of steroidogenic enzymes—StAR, p450scc, p450c17, and aromatase in the ovary on BPA exposure (Zhou et al. 2008). This supports the hypothesis that BPA exposure can cause hyperandrogenism. As the increased androgen levels activate a pro-inflammatory condition, there is a development of a pro-oxidant state in the ovaries; this, in turn, accelerates cellular apoptosis. And the hampered cellular activity due to repeated exposure of granulosa cells to low doses of BPA causes a decrease in Anti-Mullerian hormone (AMH) and estradiol levels in ovarian follicles. This could in turn lead to a decrease in ovarian reserve as it hampers the number of raised follicles and the quality of mature oocytes (Cao et al. 2018). However, the effect of BPA on the expression of *AMH* has been observed to vary with the stage of development. In the pre-pubertal period, increased levels of AMH and decreased number of corpus luteum result in a significant decrease in serum levels of P4, displaying a disruption in follicular development (Li et al. 2014).

The dysregulated functioning of the HPO-axis plays a major role in the underlying mechanism of altered hormone levels and ovarian morphology. The increased GnRH pulsatility and reduced GnRH-induced LH levels (Fernández et al. 2009) on high-dose BPA exposure further result in the alteration of sex hormone levels where serum Testosterone (T) and E2 levels are increased, and P levels are decreased (Fernández et al. 2010). This correlates the effects of BPA exposure to the altered hypothalamic-pituitary functioning as observed in PCOS.

A study conducted by Gámez et al. (2015) observes increased LH and E2 levels; this contradicts the previous results, and this can be attributed to the difference in BPA dosage and the time of exposure. Other than hyperandrogenism, BPA too has been observed to have the potential to induce oxidative stress that may affect the alteration in hormone levels (increase in T; decrease in E2, P, LH, and FSH) because of the impaired HPO-axis functioning and altered histopathology of the ovary (Ijaz et al. 2020).

## Ovarian morphological changes caused by BPA

As discussed in the previous sections, BPA has a mild estrogenic activity similar to E2 and possesses the ability to disrupt the HPO-axis. This disruption causes clinical manifestations in the form of hormonal imbalance and subsequent alteration in ovarian morphology. Various studies that focus on this aspect are summarized in Table 4.

**Table 4:** Ovarian morphological changes observed in animal models

Sl. no	Organism	Treatment dosage	Type of follicle	No. of follicle (mean/mean ± standard deviation)		Results	Reference
				Treatment group	Control group		
1	Female Sprague Dawley rats	0.625 mg kg^−1^ body weight (BPA5)	Oocytes	12	12	1. Number of oocytes decreases in a dose-dependent manner, though BPA5 displays no change 2. Animals exposed to BPA500 showed a lower number of corpus luteum and a higher number of atretic follicles, many of which were cystic 3. Both BPA-treated groups showed lower numbers of antral follicles. And the BPA500 animals had a lower total number of structures	Fernández et al. (2010)
		6.25 mg kg^−1^ body weight (BPA50)	Corpus luteum	14	15		
			Antral follicles	9	13		
			Atretic follicles	5	3		
			Preovulatory follicles	3	2		
			Oocytes	11	12		
		62.5 mg kg^−1^ body weight (BPA500)	Corpus luteum	2	15		
			Antral follicles	5	13		
			Atretic follicles	7	3		
			Preovulatory follicles	3	2		
			Oocytes	0	12		
2	Female Sprague Dawley rats	0.05 mg kg^−1^ body weight	Primary follicles	2.83±0.58	2.75±0.43	1. Rats in all groups showed normal ovarian morphology, characterized by all stages of follicular development and the presence of numerous healthy corpus luteum 2. The rats exposed to BPA had more primary and secondary follicles than those in the control group	Qiu et al. (2020)
			Secondary follicles	13.83±1.30	10.08±1.14		
			Antral follicles	3.33±0.43	2.83±0.34		
			Corpus luteum	3.91±0.63	3.17±0.44		
		0.5 mg kg^−1^ body weight	Primary follicles	3.92±0.42	2.75±0.43		
			Secondary follicles	14.67±1.75	10.08±1.14		
			Antral follicles	3.17±0.42	2.83±0.34		
			Corpus luteum	3.75±0.60	3.17±0.44		
		5 mg kg^−1^ body weight	Primary follicles	3.50±0.60	2.75±0.43		
			Secondary follicles	13.25±1.22	10.08±1.14		
			Antral follicles	3.00±0.48	2.83±0.34		
			Corpus luteum	3.33±0.51	3.17±0.44		
		10 mg kg^−1^ body weight	Primary follicles	4.50±0.63	2.75±0.43		
			Secondary follicles	18.17±1.78	10.08±1.14		
			Antral follicles	3.08±0.29	2.38±0.34		
			Corpus luteum	3.33±0.38	3.17±0.44		
3	Female CD-1 mice	12.5 mg kg^−1^ body weight	Primordial follicles	7.75	7.5	1. The number of antral follicles in the BPA-treated groups was lower than that in the control group, but this was not significant 2. The numbers of primordial follicles, primary follicles, and corpus luteum were significantly lower in the 25 and 50 mg/kg BPA-treated groups compared with the control group	Zhu et al. (2018)
			Primary follicles	8.75	8.75		
			Antral follicles	16	17.5		
			Atretic follicles	2	2		
			Corpus luteum	6	7.5		
		25 mg kg^−1^ body weight	Primordial follicles	6	7.5		
			Primary follicles	5.75	8.75		
			Antral follicles	14	17.5		
			Atretic follicles	4.5	2		
			Corpus luteum	4	7.5		
		50 mg kg^−1^ body weight	Primordial follicles	4	7.5		
			Primary follicles	4	8.75		
			Antral follicles	13	17.5		
			Atretic follicles	8	2		
			Corpus luteum	1.5	7.5		
4	Female Wistar rats	3 μg kg^-1^ day^-1^	Primary follicles	9.75	3.5	1. Significant increase in the total number of follicles is observed in the case of BPA exposure 2. The number of primary and secondary follicles is high in the BPA exposed group, as the number of antral follicles has reduced. Lastly, there is a significant increase in number, observed in the case of atretic follicles	Gámez et al. (2015)
			Secondary follicles	8	4		
			Antral follicles	7	9.5		
			Atretic follicles	7.75	4.4		
5	Female Long Evans rats	50 μg kg^-1^ (low dose)	Corpus luteum	9.75	12.43	1. BPA treated groups displayed abnormal folliculogenesis, as they contained hemorrhagic and degenerated follicles 2. The high-dose BPA treated group is least likely to progress to ovulation as observed from the significantly decreased number of corpus luteum	Adewale et al. (2009)
		50 mg kg^-1^ (high dose)	Corpus luteum	2.1	12.43		
6	Female Sprague Dawley rats	50 μg kg^-1^ (BPA1)	Corpus luteum	12.0 ± 0.31	12.6 ± 0.24	1. Significant decrease was observed in the number of corpus luteum in BPA2, BPA3, and BPA4 groups, in comparison to the control group 2. A significant increase in the number of antral follicles and atretic follicles were observed in BPA3 and BPA4 group 3. No significant results were observed in the case of preovulatory follicles across all BPA-treated groups	Ijaz et al. (2020)
			Antral follicles	11.8 ± 0.20	10.6 ± 0.24		
			Atretic follicles	1.6 ± 0.24	2.2 ± 0.37		
			Preovulatory follicles	2.2 ± 0.37	2.0 ± 0.31		
		500 μg kg^-1^ (BPA2)	Corpus luteum	10.8 ± 0.49	12.6 ± 0.24		
			Antral follicles	10.8 ± 0.37	10.6 ± 0.24		
			Atretic follicles	2.8 ± 0.20	2.2 ± 0.37		
			Preovulatory follicles	2.6 ± 0.40	2.0 ± 0.31		
		5 mg kg^-1^ (BPA3)	Corpus luteum	10.6 ± 0.40	12.6 ± 0.24		
			Antral follicles	8.8 ± 0.37	10.6 ± 0.24		
			Atretic follicles	4.4 ± 0.24	2.2 ± 0.37		
			Preovulatory follicles	2.2 ± 0.37	2.0 ± 0.31		
		50 mg kg^-1^ (BPA4)	Corpus luteum	6.4 ± 0.24	12.6 ± 0.24		
			Antral follicles	6.8 ± 0.58	10.6 ± 0.24		
			Atretic follicles	6.8 ± 0.80	2.2 ± 0.37		
			Preovulatory follicles	1.8 ± 0.37	2.0 ± 0.31		

*BPA* bisphenol A, μg microgram, kg kilogram, mg milligram

A dose-dependent decrease in oocytes is observed with an increase in the dose of BPA exposure because of oocyte apoptosis (Fernández et al. 2010). This in turn shows lowered development of primordial follicles on exposure to high doses of BPA (Zhu et al. 2018). On BPA exposure the alteration in expression of *ESR1* and *ESR2* in ovarian follicles causes an increase in follicular recruitment (Rodríguez et al. 2010), thereby resulting in the observation of increased numbers of preovulatory follicles (Fernández et al. 2010; Ijaz et al. 2020), primary follicles (Gámez et al. 2015), and secondary follicles (Gámez et al. 2015). It has been observed that BPA alters follicular growth and induces atresia, thereby leading to observations of increased atretic follicles (Fernández et al. 2010; Gámez et al. 2015; Ijaz et al. 2020) on exposure to high doses. This in turn leads to decreased number of antral follicles (Fernández et al. 2010; Gámez et al. 2015; Ijaz et al. 2020; Zhu et al. 2018), hence increasing rates of follicular arrest that leads to anovulation (Rutkowska and Rachoń 2014). As the BPA-exposed animals exhibit reduced LH secretion because of the irregular GnRH pulsatility (Fernández et al. 2009), its ability to cause cell enlargement and production of P may decrease (Azziz et al. 2016), and this paired with low numbers of antral follicles that may mature leads to a significant decrease in the number of corpus luteum observed (Fernández et al. 2010; Ijaz et al. 2020; Zhu et al. 2018; Adewale et al. 2009).

A study conducted by Qiu et al. (2020) observes normal ovarian morphology and follicular development, in adult rats that were neonatally exposed to BPA. Since the time of exposure plays a huge role in the development of effects, this could contribute to the difference in results (Qiu et al. 2020).

## Effect of BPA on PCOS

The effect of BPA on pathways like insulin signaling, lipid metabolism, ovarian steroidogenesis, and the functioning of the HPO-axis is well observed in animal models and in vitro human cell line studies. The results obtained can be correlated with the findings in various human epidemiology studies. These studies focus on the relationship between BPA and the factors crucial in the clinical manifestation and pathogenesis of PCOS, by establishing a correlation between the presence of BPA and the altered levels of hormone levels in PCOS patients.

BPA has been observed to cause disruptions in several metabolic pathways and functioning of the endocrine system, thereby giving rise to PCOS in premenopausal women. Since BPA mimics the activity of 17-β estradiol, it has been reported to possess the ability to disrupt steroid feedbacks at the hypothalamus-pituitary level and steroid action at the ovarian level, thereby suppressing HPO-axis functions (Wang et al. 2017). This includes hypersecretion of circulating LH and increased levels of FSH, causing alteration of LH:FSH ratios (Vahedi et al. 2016).

In addition, BPA contributes to the disarrayed metabolic profile in PCOS due to the involvement of BPA in insulin resistance (Kandaraki et al. 2011) and its promotion of inflammatory conditions through the development of obesity and infiltration of macrophages into the adipose tissue (Tarantino et al. 2013). Chronic inflammation aids the development of insulin resistance, and the subsequent compensatory hyperinsulinemia indirectly leads to increased amplitude and frequency of GnRH and LH pulse secretion observed in PCOS. This increase also induces relative resistance of follicles to FSH and subsequent increase in production of AMH, promoting a decrease in antral follicle count thereby plausibly impairing the ovarian reserve (Zhou et al. 2017). Furthermore, BPA exposure causes attenuation of aromatase expression in the follicular fluid causing dysregulation in estrogen production (Wang et al. 2017). This paired with the potential of BPA to increase androgen levels due to its inhibitory effect on the action of testosterone-hydroxylase (Takeuchi et al. 2006) has the ability to cause hyperandrogenism (Jurewicz et al. 2021). 

**Fig. 2:**
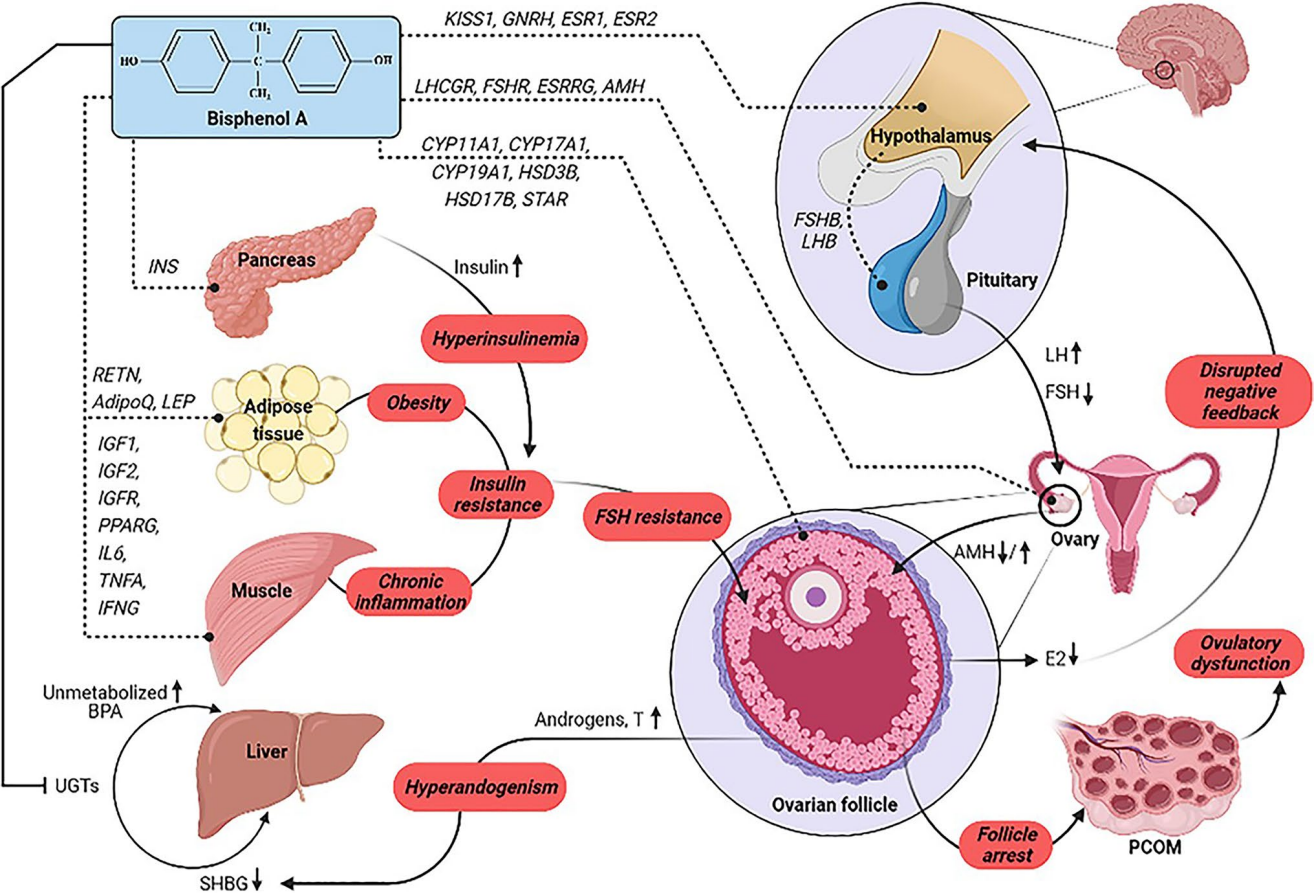
The effect of BPA on the expression of certain genes involved in the PCOS pathogenesis

Moreover, a prospective observational study conducted by Déchaud et al. (1999) suggests that an increase in free testosterone levels in the serum is because of the ability of BPA to displace sex steroid hormones from SHBG. In association with these findings, it appears that in PCOS the metabolism and excretion of BPA are impaired due to the effect of hyperandrogenism in blocking the expression of uridine diphosphate-glucuronosyl transferase (UGTs) that completely metabolizes BPA (Vermeulen 1993). The resulting effects are well studied by Takeuchi et al. (2004) in a case-controlled study that indicates increased BPA levels in women diagnosed with PCOS. Despite BPA having a short half-life, it has an inhibitory effect on the activity of UGT, which causes BPA to remain incompletely metabolized thereby increasing its levels in the body. This causes an alteration in gene expression that disturbs various endocrinal processes like gonadotropin secretion and receptivity, ovarian steroidogenesis, insulin activity, and regulation of adipokines, which in turn causes demonstration of clinical manifestations like ovulatory dysfunction, disrupted folliculogenesis, polycystic ovarian morphology, hyperandrogenism, hyperinsulinemia, obesity that are associated with PCOS (Fig. 2).

All these results compiled suggest that the presence of BPA in women with PCOS could be both a cause and consequence of disrupted hormonal patterns and metabolic activity that result in clinical manifestations like hyperandrogenism, ovulatory dysfunction observed as the length of the menstrual cycle and duration of bleeding (Rashidi et al. 2017) and altered ovarian morphology.

## Conclusion

Since the studies on gene expression in BPA-induced PCOS models are relatively limited, this review encapsulates the effect of BPA on the expression of 28 genes that take part in various metabolic and endocrinal pathways, whose disruption may play a major role in the pathogenesis of PCOS.

## Data Availability

Not applicable.
